# Maternal satisfaction on delivery care services and associated factors at public hospitals in eastern Ethiopia

**DOI:** 10.1093/inthealth/ihac038

**Published:** 2022-06-07

**Authors:** Addisalem Kidane, Tamirat Getachew, Firehiwot Mesfin, Addis Eyeberu, Merga Dheresa

**Affiliations:** School of Nursing and Midwifery, College of Health and Medical Sciences, Haramaya University, Harar, Ethiopia; School of Nursing and Midwifery, College of Health and Medical Sciences, Haramaya University, Harar, Ethiopia; School of Public Health, College of Health and Medical Sciences, Haramaya University, Harar, Ethiopia; School of Nursing and Midwifery, College of Health and Medical Sciences, Haramaya University, Harar, Ethiopia; School of Nursing and Midwifery, College of Health and Medical Sciences, Haramaya University, Harar, Ethiopia

**Keywords:** client satisfaction, delivery service, healthcare, mothers

## Abstract

**Background:**

Maternal healthcare services satisfaction has been widely recognized as a critical indicator of quality in healthcare systems. Thus this study aimed to assess maternal satisfaction with delivery care services.

**Methods:**

An institutional-based cross-sectional study design was utilized among 400 randomly selected postnatal mothers from 1 to 30 February 2018. The data were entered into EpiData version 4.2.0 and computed using SPSS version 20. Bivariate and multivariate analyses were done using binary logistic regression to identify associations of factors.

**Results:**

A total of 400 participants were included, with a response rate of 98.8%. The overall delivery services satisfaction level of mothers was 80% (95% confidence interval [CI] 75.8 to 84.0). Delivery through caesarean section (adjusted odds ratio [AOR] 2.85 [95% CI 1.21 to 6.72]), privacy assured (AOR 3.73 [95% CI 1.79 to 7.75]), duration of labour (AOR 3.03 [95% CI 1.50 to 6.14]), waiting time (AOR 4.31 [95% CI 2.24 to 8.29]) and foetal outcome (AOR 4.33 [95% CI 1.94 to 9.66]) were associated with satisfaction with delivery care services.

**Conclusion:**

The study revealed that four-fifths of mothers were satisfied with the delivery care services provided in public hospitals. Much effort is needed from hospital administrators and health professionals to improve delivery services satisfaction by minimizing waiting time, maintaining privacy and securing waiting areas.

## Introduction

Satisfaction with delivery care services is a means of secondary prevention of maternal mortality, since satisfied women may be more likely to adhere to healthcare providers’ recommendations.^1^ Satisfaction is defined as the state of pleasure or contentment with an action, event or service and is determined by clients’ expectations and experiences.

Maternal satisfaction with the services provided during delivery has been recognized as a critical indicator of the quality of a healthcare system. Satisfied clients have a higher chance of returning to the facility in the future and of recommending the institution to their neighbours and relatives.^2,3^

A positive childbirth experience and a favourable attitude towards motherhood will enhance women's satisfaction with the care provided during labour and delivery, which facilitates a positive transition into the maternal role. On the other hand, a negative birth experience can cause the woman to feel distraught and harm her mental health, which increases the risks of postpartum blues, depression, psychotic disorders and post-traumatic stress disorder.^4,5^

Similarly, a mother who is not satisfied with delivery services will avoid hospital delivery for her next pregnancy and may prefer home delivery with traditional birth attendants, which leads to maternal mortality due to sepsis and postpartum haemorrhage. Negative experiences, including traumatic birth, could lead to memories of pain, anger and fear that may predispose mothers to postpartum depression, extreme distress and even neglect and abuse of children .^6^

Maternal mortality can be linked to delays in receiving care, inadequate skilled personnel in emergency obstetric care, inadequate supplies and equipment and poor quality of services. Understanding maternal perceptions of care and satisfaction with services is important in this regard, as perceived quality is a key factor affecting service utilization.^7^

The Ethiopian government aimed to reduce maternal mortality from 412 to 199 per 100 000 live births and a set of high-impact interventions were implemented for free, including antenatal care (ANC), skilled birth services and postnatal care (PNC). However, the existence of free delivery healthcare services neither promises their utilization by women nor does it guarantee optimal pregnancy outcomes or satisfaction with the care services. Long waiting times, unavailability of basic drugs and poor physical environment of the healthcare facilities were identified as the major factors for mothers’ dissatisfaction in Ethiopia.^8–11^

Maternal satisfaction was influenced by several factors, but there are many disagreements among researchers because these factors were reported differently from study to study. Evidence shows that satisfaction with care boosts women's sense of accomplishment and self-esteem, heightens their expectation of future positive childbirth experiences and promotes breastfeeding and bonding with their children.^6^

Despite the implementation of compassionate, respectful and caring health services by the Ethiopian Federal Ministry of Health, the level of maternal satisfaction with delivery services at public hospitals remains poorly addressed. In Ethiopia, women do not have confidence in the quality of public hospital services. However, little is known about maternal satisfaction; almost all previous studies are either qualitative studies or measure satisfaction levels using a simple yes/no questionnaire^12,13^ in which predictors are not well addressed. Therefore this study tried to overcome this gap by using a Likert scale questionnaire through independent observation of structural- and process-related predictors. Thus we aimed to assess maternal satisfaction with delivery care services at public health hospitals in eastern Ethiopia.

## Methods

### Study area and period

The study was conducted at public hospitals in the Harari regional state in eastern Ethiopia. Harar is the capital city of the Harari regional state, which is 518.19 km from the capital city of Ethiopia, Addis Ababa. The Harari region has an estimated total population of 225 136, of which 58.6% are estimated to be rural inhabitants and 41.4% are urban dwellers.^14^ There are two public hospitals that provide maternal health services in the Harari region (Jugol General Hospital and Hiwot Fana Specialized University Hospital [HFSUH]) and both hospitals were included in the study. Jugol Hospital is one of the public hospitals in Harar. The hospital is run by 248 health professionals (personal communication, Jugol Hospital human resource management, 2017). The HFSUH is the largest hospital in eastern Ethiopia, built in 1928 by Italy to serve Italian soldiers, and is now being managed by Haramaya University. The hospital provides general treatment and is open 24 h for emergency services. The hospital has a total of 186 beds, with separate obstetrics ward, postnatal ward, surgical ward, medical ward, maternal and child health clinics, operating theatre, gynaecologic ward, paediatrics as an outpatient department, anti-retroviral treatment (ART) clinic, dental clinic and ophthalmology clinic. This study was conducted from 1 to 30 February 2018.

### Study design and population

An institutional-based cross-sectional study design was employed. The source population was mothers who visited public hospitals in the Harari regional state and the study population was all mothers who gave birth in Harari regional state public hospitals. Mothers who gave birth in the Harari regional public hospitals but were unable to communicate because of serious illness or impaired cognition during the data collection period were excluded from the study.

### Sample size determination and sampling procedure

The double population formula was used to compute the sample size for the study using Stat Calc in the Epi Info 7, with the assumption of power of 80%, level of significance (confidence interval [CI]) 95%, ratio of exposed to non-exposed 1:1 and percentage of exposed (<6 h labour time) 85.7% and non-exposed (>6 h labour time) 73.4%. Therefore the final sample size by adding a non-response rate of 10% became 405.

The number of study participants in each hospital was proportional to the population size by reviewing the average 3-month delivery service report of the public hospitals in the region. The study participants were selected by a consecutive sampling method and an exit interview was performed at the time mothers were discharged from the postnatal unit.

### Data collection methods

The data were collected by a structured questionnaire that has three parts. The first part asks about sociodemographic information of mothers and the second part is about obstetric factors. Finally, the satisfaction level of mothers was measured using questions that were adopted from the Donabedian Quality Assessment Framework,^15^ presented using a 5-point Likert scale ranging from ‘very dissatisfied’ to ‘very satisfied’. The first draft of the English questionnaire was translated into Amharic and Afaan Oromo by independent translators and then back to English to check for consistency. To maintain data quality, six female nurses were recruited as data collectors who spoke Amharic and Afaan Oromo and supervision was conducted by two bachelor of science (BSc) degree holder midwifery professionals who were not affiliated with the hospitals included in the study.

The data were collected during the early postpartum period through an exit interview at the time mothers were discharged from the postnatal unit. The data collectors conducted a face-to-face interview using the pretested structured Amharic and Afaan Oromo questionnaires. The interviewers informed the women about all details of the research. A brief introductory orientation was given to the study participants by data collectors about the purpose of the study. They were told about the importance of their involvement, then the mothers were interviewed face to face. At the end of each day, the questionnaires were reviewed and cross-checked for completeness, accuracy and consistency by the supervisor and principal investigator and corrective measures were taken if necessary.

### Study variables

The dependent variable was maternal satisfaction level. Independent variables included sociodemographic/economic factors (age, religion, marital status, education level, mother's occupation, residence and monthly income) and obstetric factors (parity, ANC, pregnancy status, duration of labour, mode of delivery, foetal condition and maternal outcome). Furthermore, structure-related factors (physical environment, presence of a waiting area, comfort of the waiting area, service charge, availability of laboratory services, drugs and cleanliness of the toilet) and process and care-related factors (sex of care provider, waiting time, privacy, perceived provider competency, pain management, newborn care, support from staff in breastfeeding, asking permission throughout each procedure, politeness of healthcare provider, number of healthcare providers and promptness of care) were also included as independent variables.

### Operational definitions and measurement

Maternal satisfaction is the satisfaction of mothers during service delivery. It is the care level that increases the likelihood of future utilization of maternal health services.^16^ This includes mothers’ satisfaction with the waiting time to be seen by healthcare providers, permission before doing procedures, asking permission throughout each procedure, the cost paid for the services, explanation about labour progress, helpfulness and politeness of healthcare providers, verbal encouragement and reassurance during labour, availability of caregivers, competency and confidence of healthcare providers, privacy during physical examinations, sex of healthcare providers, overall care and support, support from staff during breastfeeding, care given to the baby, pain management, number of healthcare providers, availability of drugs and medical supplies, size of rooms and beds, availability of laboratory services, sanitation of toilets and the presence and comfort of waiting areas. Those items are important indicators for maternal satisfaction with delivery care services.^17^

The level of satisfaction was assessed on a 5-point Likert scale (1, very dissatisfied; 2, dissatisfied; 3, neutral; 4, satisfied; 5, very satisfied). For overall satisfaction, those who were satisfied with ≥75% of the items were categorized as ‘satisfied’ (those who responded very satisfied, satisfied or neutral) and those who were satisfied with <75% of the items were categorized as ‘unsatisfied’ (those who responded dissatisfied or very dissatisfied).^9^

Waiting time was the time between admission and being seen by health professionals.^18,19^

Promptness of care was the provision of care without delay/immediately, with no prolonged waiting time.

Perceived provider's competency was mothers’ perspective on the ability of healthcare providers to give care successfully and efficiently.

Privacy assurance was the ability of mothers to obtain delivery care services without being observed or disturbed by other people and to be examined in a separate room.

### Data quality control

Two days of training was provided for data collectors and supervisors on how to approach and interview respondents and complete the questionnaire. On each data collection day, the collected data were reviewed and checked for missing information, legibility of handwriting, completeness and consistency and any mistake or ambiguity was noted by the principal investigator and supervisor; any problems faced at the time of data collection were discussed and an immediate solution was provided. The questionnaire was pretested on 20 mothers at Dilchora Hospital in Diredawa before the actual data collection to determine the accuracy of responses, language clarity and appropriateness of the tools. The necessary changes were made based on the findings of the pretest. The amended tool was then used for data collection at the selected health facilities.

### Methods of data processing and analysis

The data were coded, cleaned, edited and entered into EpiData version 4.2.0 to minimize logical errors and design skipping patterns. Then the data were exported to SPSS for Windows version 20 (IBM, Armonk, NY, USA) for analysis. Maternal satisfaction with delivery services was considered as a dependent variable. To measure maternal satisfaction, a 21-item 5-point Likert scale with a score range of 21–105 was used. Descriptive analysis was done by computing proportions and summary statistics. Simple frequencies, summary measures, tables and figures were used to present the data. Multicollinearity was checked using the variance inflation factor (VIF) and standard error (SE) and variables with a SE >2 or a VIF >10 were dropped. The goodness of fit was tested by Hosmer–Lemeshow and Omnibus tests. The model was considered a good fit since it was found to be insignificant for the Hosmer–Lemeshow statistic (p=0.672) and significant for the Omnibus test (p≤0.001). The association between each independent variable and the outcome variable was observed by binary logistic regression. The direction and strength of statistical associations were measured by the odds ratio (OR) with 95% CI. Adjusted ORs along with 95% CIs were estimated to identify factors associated with mothers’ satisfaction by using multivariate analysis in the binary logistic regression. Finally, statistical significance was declared at p<0.05.

### Ethical considerations

Ethical clearance was obtained from Haramaya University, College of Health and Medical Sciences, Institutional Health Research Ethics Review Committee (IHRERC). A formal letter of permission and support was written to each respective hospital from Haramaya University. All the study participants were informed about the purpose of the study and their right to refuse or discontinue the interview at any time. Informed voluntary, written and signed consent was obtained from each study participant before the interview. They were also informed that the information obtained from them would be treated with complete confidentiality. All methods used throughout the study were carried out per Haramaya University guidelines and regulations.

## Results

### Sociodemographic characteristics

A total of 400 of 405 study participants were involved (response rate 98.8%). The mean age of the participants was 25.97 y (standard deviation [SD] 5.399). Almost all participants (387 [96.8%]) were married and 174 (43.5%) were housewives. More than two-thirds (264 [66.0%]) of the respondents lived in an urban area. A total of 181 (45.3%) participants had no formal education and 252 (63.0%) had a monthly income ≥1500 birr (∼US$1.9/day) based on the current Ethiopian poverty baseline (Table 1).

**Table 1.** Sociodemographic characteristics of postnatal mothers, 2018 (N=400)

Variables		n	%
Maternal age (years)	<20	86	21.5
	21–34	276	69.0
	35–49	38	9.5
Marital status	Married	387	96.8
	Other	13	3.2
Religion	Muslim	267	66.8
	Orthodox	107	26.8
	Other	26	6.5
Ethnicity	Oromo	207	51.8
	Amhara	105	26.3
	Hadere	33	8.3
	Other	55	13.8
Level of education	No formal education	181	45.3
	Primary education	95	23.8
	Secondary education	87	21.8
	College and above	37	9.3
Occupation	Housewife	174	43.5
	Private employee	72	18.0
	Farmer	52	13.0
	Government employee	47	11.8
	Other	55	13.8
Residence	Urban	264	66.0
	Rural	136	34.0
Monthly income (birr)	≤1500	148	37.0
	>1500	252	63.0

### Obstetrics characteristics of the respondents

Of the respondents, nearly half (192 [48%]) were primiparous. A total of 182 (45.5%) said others had recommended them to deliver at a hospital. Duration of labour was <12 h in 323 (80.7%) of the respondents. Around three-fourths of mothers (301 [75.2%]) had an ANC follow-up and 224 (74.4%) had less than four ANC visits (Table 2).

**Table 2.** Obstetrics characteristics of respondents (N=400)

	Overall satisfaction		
Variables	Satisfied, n (%)	Unsatisfied, n (%)	Total, n (%)
Parity			
Primipara	163 (84.9)	29 (15.1)	192 (48.0)
Multipara	138 (78.9)	37 (21.1)	175 (43.7)
Grand multipara	19 (57.6)	14 (42.4)	23 (8.3)
Institution preference			
Recommended by others	146 (80.2)	36 (19.8)	182 (45.5)
Satisfied with the previous birth	68 (76.4)	21 (23.6)	89 (22.3)
Referral	106 (82.2)	23 (17.8)	129 (32.2)
Current pregnancy status			
Wanted	287 (81.5)	65 (18.5)	352 (88.0)
Unwanted	33 (68.7)	15 (31.3)	48 (22.0)
Length of labour (hours)			
≤12	266 (82.4)	57 (17.6)	323 (80.7)
>12	54 (70.0)	23 (30.0)	77 (19.3)
Mode of delivery			
SVD	212 (77.0)	63 (23.0)	275 (68.7)
Instrument delivery	23 (74.2)	8 (25.8)	31 (7.8)
CS	85 (90.4)	9 (9.6)	94 (23.5)
Assure privacy			
No	36 (64.3)	20 (35.7)	56 (14.0)
Yes	284 (82.5)	60 (17.5)	344 (86.0)
Waiting time (minutes)			
>15	165 (72.7)	62 (27.3)	227 (56.8)
≤15	155 (89.6)	18 (10.4)	173 (43.2)
Foetal condition			
Normal	283 (86.8)	43 (13.2)	326 (81.5)
With complication	34 (63.0)	20 (37.0)	54 (13.5)
Died	3 (15.0)	17 (85.0)	20 (5.0)
ANC follow-up			
Yes	235 (78.0)	66 (22.0)	301 (75.2)
No	85 (85.8)	14 (14.2)	99 (24.8)
Number of ANC visits (n=301)			
<4	189 (84.4)	35 (15.6)	224 (74.4)
≥4	50 (65.0)	27 (35.0)	77 (25.6)

### Women's satisfaction with delivery services

**Figure 1.**
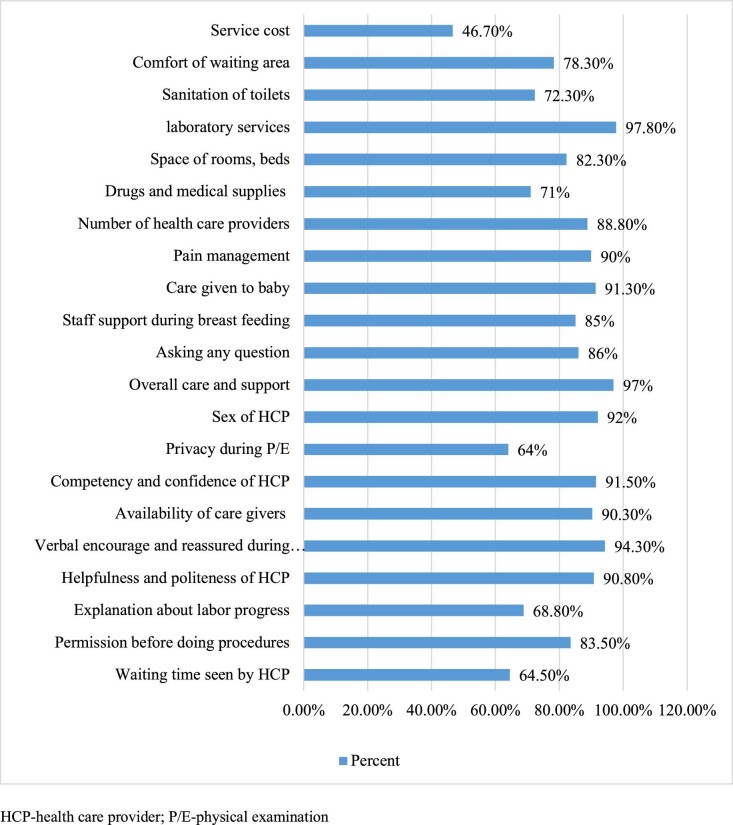
Mother's satisfaction with the components of maternal satisfaction, 2018 (N=400).

In this study, the overall satisfaction level of mothers with delivery service was 80% (95% CI 75.8 to 84.0). The structure- and process-related satisfaction of mothers was 62.3% (95% CI 57.3 to 67.2) and 81.3% (95% CI 77.8 to 84.8), respectively. The availability of laboratory services (391 [97.8%]), overall care-related satisfaction (388 [97%]), verbal encouragement and reassurance during labour (377 [94.3%]) and the sex of healthcare providers (368 [92%]) were the first four listed values among the satisfaction assessment items (Figure 1).

**Figure 2.**
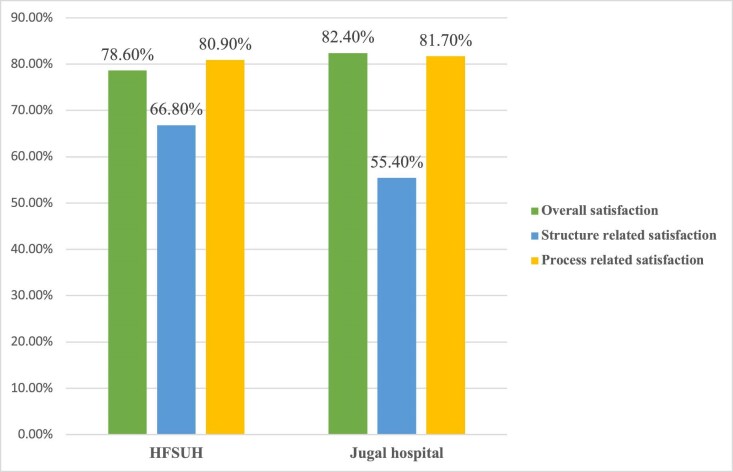
Mother's satisfaction with delivery services in public hospitals in Harari region, Ethiopia, 2018 (N=400).

Concerning the satisfaction level for each hospital, the overall satisfaction level of women was 82.4% for Jugal Hospital and 78.6% for HFSUH. Process-related satisfaction was almost equal in both hospitals: 80.9% for HFSUH and 81.7% for Jugal Hospital (Figure 2).

### Factors associated with delivery service satisfaction

Mothers who delivered through caesarean section (CS) were 2.85 times more likely to be satisfied than those who delivered through spontaneous vaginal delivery (SVD) (adjusted odds ratio [AOR] 2.85 [95% CI 1.21 to 6.72]). Mothers whose privacy was assured were 3.73 times more likely to be satisfied than mothers whose privacy was not assured (AOR 3.73 [95% CI 1.79 to 7.75]). Regarding the duration of labour, those whose labour persisted for ≤12 h were three times more likely to be satisfied compared with women whose labour persisted >12 h (AOR 3.03 [95% CI 1.50 to 6.14]).

Those participants who waited ≤15 min to be seen by the healthcare provider were about four times more likely satisfied compared with women who waited >15 min (AOR 4.31 [95% CI 2.24 to 8.29]). Those respondents whose foetal condition was normal were 4.33 times more likely satisfied compared with women whose foetus was delivered with a complication (AOR 4.33 [95% CI 1.94 to 9.66]) (Table 3).

**Table 3.** Factors associated with mother's satisfaction with delivery services in public hospitals, 2018 (N=400)

Variables	Unsatisfied, n (%)	Satisfied, n (%)	Crude OR (95% CI)	AOR (95% CI)
Mother’s educational status				
College and above	12 (32.5)	25 (67.5)	1	1
Secondary education	22 (77.3)	65 (22.7)	1.41 (0.61 to 3.28)	0.97 (0.36 to 2.61)
Primary education	15 (15.8)	80 (84.2)	2.56 (1.06 to 6.18)	2.45 (0.90 to 6.72)
No formal education	31 (17.2)	150 (82.8)	2.32 (1.05 to 5.11)	1.70 (0.59 to 4.85)
Residence				
Urban	63 (24.0)	201 (76.0)	1	1
Rural	17 (12.5)	119 (87.5)	2.19 (1.22 to 3.92)	1.59 (0.62 to 4.06)
Maternal age (years)				
35–49	10 (26.3)	28 (73.7)	1	1
25–34	57 (20.7)	219 (79.3)	1.37 (0.63 to 2.98)	1.80 (0.67 to 4.81)
≤24	13 (15.2)	73 (84.8)	2.01 (0.78 to 5.09)	2.74 (0.88 to 8.49)
Duration of labour (hours)				
≥12	23 (30.0)	54 (70.0)	1	1
<12	57 (17.7)	266 (82.3)	1.98 (1.12 to 3.50)	3.03 (1.50 to 6.14)*
Waiting time (minutes)				
>15	62 (27.3)	165 (72.7)	1	1
≤15	18 (10.4)	155 (89.6)	3.23 (1.83 to 5.71)	4.31 (2.24 to 8.29)**
Condition of foetus				
With complication	20 (35.2)	34 (64.8)	1	1
Normal	61 (17.7)	285 (82.3)	2.75 (1.36 to 4.73)	4.33 (1.94 to 9.66)**
Assure privacy				
No	20 (35.7)	36 (64.3)	1	1
Yes	60 (17.5)	284 (82.5)	2.63 (1.42 to 4.85)	3.73 (1.79 to 7.75)**
ANC follow-up				
Yes	66 (22.0)	235 (78.0)	1	1
No	14 (14.2)	85 (85.8)	1.70 (0.91 to 3.19)	2.13 (0.89 to 5.10)
Mode of delivery				
SVD	63 (33.0)	212 (77.0)	1	1
Instrument delivery	8 (25.8)	23 (74.2)	0.85 (0.36 to 2.00)	0.70 (0.26 to 1.92)
CS	9 (9.6)	85 (90.4)	2.80 (1.33 to 5.89)	2.85 (1.21 to 6.72)**
Pregnancy status				
Unwanted	15 (31.3)	33 (68.7)	1	1
Wanted	65 (18.5)	287 (81.5)	2.01 (1.03 to 3.91)	2.28 (0.98 to 5.25)

Significant at *p=0.05, **p<0.001 and 1=reference.

## Discussion

This study revealed that the overall satisfaction level with delivery service in two dimensions was 80%. The structure-related and process-related satisfaction of mothers was 62.3% and 81.3%, respectively. The mode of delivery, assured privacy, duration of labour, waiting time and foetal condition were factors associated with the mother's satisfaction with delivery services.

This implies that about 20% of respondents are prone to give birth at home because of the dissatisfaction arising from the service given at public hospitals. However, the trend of institutional deliveries has increased from 5% in 2000 to 26% in 2016, according to the Ethiopian Demographic and Health Survey.^14^ Being dissatisfied with healthcare services may result in a maternal preference for home delivery, which worsens the rate of maternal mortality and morbidity in the country. Moreover, being dissatisfied with the service from public facilities causes mothers to pay unplanned fees for delivery at private institutions.

The overall satisfaction with delivery service was 80%. This finding was consistent with the study conducted in Debre Markos (81.7%), Assela Hospital (80.7%), Mekelle (79.7%), Wolaita Zone (82.9%) and Jimma University Specialized Hospital (77%).^9,10,17,21^ This was higher than in the study conducted in Felege Hiwot Referral Hospital, Ethiopia (74.9%)^21^ and Nairobi, Kenya (56%),^22^ but was lower than in the study conducted in Arbaminch district, southern Ethiopia (90.2%). The possible reason for better maternal satisfaction in this study might be due to the implementation of a compassionate and respectful maternal service.^23^ This could be improving the quality of maternal health services with the involvement of different stakeholders, including nurses, midwives and obstetricians. It also might be because of a difference in the quality of services provided, the expectations of mothers or the types of health facilities (the study was conducted in urban public hospitals and did not include health centres and private health institutions). Another justification might be Likert scale usage in measuring maternal satisfaction levels through independent observations of structure- and process-related factors.

Mothers who delivered through CS were more likely to be satisfied with delivery service than mothers who delivered through SVD. This was in line with studies conducted in Debre Markos^9^ and Gamo Gofa Zone, southwest Ethiopia.^16^ This could be because those who delivered through SVD and instrument delivery might be injured or suffered from labour pain; they might also fear the complications related to instrument delivery. However, those who gave birth by CS get relief by anaesthesia from labour pain and the operation results in a better neonatal outcome, especially in a distressed foetus. Moreover, healthcare providers prioritized the distribution of limited supplies, including close follow-up, to operative births to prevent infection and related complications.^11^

Mothers whose privacy was assured were more likely to be satisfied than their counterparts. This was in line with the study conducted in referral hospitals of Amhara region,^24^ Assela Hospital^17^ and Mekelle.^25^ This may be because mothers’ have a greater chance to communicate with healthcare providers regarding the need for labour pain analgesia and receive better reassurance and counselling. On the other hand, since the study included a teaching hospital, the delivery process might be attended by more than two individuals, including students who are observing the procedure for academic purposes. Unless the mother is informed about the experience and skill level of delivery attendants, the mother might worry that she is going to be attended by students. Therefore, wherever the delivery place is, mothers need great privacy and respect in a sociocultural context.

Pain during labour and childbirth is unique and the most severe pain event in a woman's life. The study revealed that those mothers whose labour persisted for ≤12 h were more likely satisfied compared with women whose labour persisted for >12 h. This was in line with the studies conducted in Debre Markos^9^ and Wolaita.^20^ This might be because labouring for a longer duration may cause exhaustion and expose the woman to repeated obstetric procedures like vaginal examinations,^26,27^ and as time elapses the level of stress about the birth outcome might also be increased,^28^ resulting in less maternal satisfaction. Similarly, in this study, it was found that a breach in privacy was associated with mothers’ dissatisfaction. This was consistent with a previous study.^26^

Those participants who waited ≤15 min to be seen by the healthcare provider were more likely satisfied compared with women who waited for >15 min. This was similar to the studies conducted in Nigeria,^29^ St. Paul's Hospital Millennium Medical College,^30^ referral hospitals of Amhara region Ethiopia^24^ and Assela Hospital.^17^ This might be because the long waiting period resulted in dissatisfaction due to the absence of reassurance from the healthcare provider, failure to provide labour pain management and poor or absent waiting areas.

Those respondents whose foetal outcome was normal were more likely to be satisfied compared with women whose foetuses were delivered with complications. This was similar to the study conducted in Amhara region referral hospitals.^24^ This might be because pregnant mothers and family members wish and expect to have a normal baby, so if they lose their baby or have a baby with a complication, the mother may believe that it is the fault of the healthcare professional who attended her or the hospital in general, resulting in reduced trust to deliver at a health facility. This may later hinder governmental efforts towards decreasing child and maternal mortality by promoting home delivery.

### Limitations

The data are restricted to the delivery experiences of the hospitals chosen for the study, thereby limiting generalization to the overall health facility experience of childbirth. Potential response bias is often present in patient satisfaction studies related to social desirability bias. We tried to minimize this bias by interviewing mothers in a separate room using trained midwifery professionals who were not affiliated with the facilities studied. Even though there is a difference in traumatic childbirth experiences among mothers who undergo emergency and elective CS, our data fail to assess these two variables independently. This may conceal the true difference in delivery service satisfaction between the mothers who had deliveries through emergency and elective CS.

## Conclusions

Generally, four-fifths of mothers were satisfied with the overall care provided in the public hospitals. The mode of delivery, assured privacy, duration of labour, waiting time and foetal outcome were factors associated with the mother's satisfaction with delivery services. Health facilities are expected and encouraged to supervise the delivery service for mothers to provide compassionate and respectful maternity care in order to decrease maternal and child mortality and morbidity. Much effort is needed from hospital administrators and health professionals to improve delivery services satisfaction and minimize the preference of home delivery through shorter wait times, maintaining privacy and providing secure and comfortable waiting areas.

## Data Availability

Pertinent data are presented in this article. Additional data can be requested from the corresponding author.
